# Mr. Wagstaffe's Case of Vaccine

**Published:** 1803-06-01

**Authors:** I. F. Wagstaffe

**Affiliations:** Tooley Street


### *To the Editors of the Medical and Physical Journal.*

GENTLEMEN,

I DID not intend mentioning my opinion at present of the causes of fatality in the two children whom I inoculated with genuine Vaccine fluid, and whose cases are concisely stated in your last Journal, had it not been that some of the friends of the new Inoculation appear somewhat alarmed, lest it should excite an unfavorable opinion of its effects on peculiar constitutions; I therefore feel it a duty incumbent on me to remove any doubt which may arise from such an idea.

From the conversation I had with several medical gentlemen at the time of these cases existing, I could not find they had ever met with a similar occurrence. This I consider as a very strong argument that the illness was wholly independent of Vaccine influence. I thought it was probable some of your correspondents would notice the cases, and make some comments thereon, which would have produced a reply from me according to their remarks; and if it should appear that such have not occurred to others, it would tend much to strengthen the opinion, that the Vaccine disease had no connection with the convulsions, or their effects.

It was the opinion of those gentlemen to whom I related the cases, that the affection of the stomach and intestines, to which the convulsions succeeded, was that kind of accidental attack which would equally have produced its effect if the children had not been under the influence of Vacciola. The short period of existence after the attack in the *first* child, precluded that investigation which could have been wished.

During a period of six weeks, the second child had convulsions daily, but was at intervals sensible; the diarrhœa and vomiting were but slightly restrained during the whole subsequent illness; the pustule on the arm dried up but never fell off. I wished much to have inspected the body, but could not prevail on the parents to permit me.

At first I found the parents somewhat inclined to attribute the convulsions to the effect of Vaccination, which I strenuously opposed, and ultimately convinced them, that no connexion existed between the two diseases.

Convulsion, as an idiopathic disease, has been considered as one of the most frequent to which children are subject, though though I believe more have been considered such than facts would warrant.

As a symptomatic disease, convulsions are very frequent. Dr. Underwood, in his Treatise on the diseases of children, says (vol. i. page 159) "The younger and *more irritable* an infant may be, it will be so much the more liable to *symptomatic* convulsion, especially from any considerable *disturbance in the first passages*.

It was very evident the attack in each of these cases commenced in the primæ viæ; the whole alimentary canal was thrown into violent spasmodic action, first the stomach and then the intestines. In the first child this irritation was so great as to excite immediate convulsions, soon terminating its existence. In the second, though the attack was equally violent, the constitution was capable of resisting its effects a greater length of time.

I should therefore infer, that the convulsions were produced by the morbidly increased action of the primæ viæ, and that those morbid actions were induced either by the presence of accumulation in the stomach or by the specific action of miasma. I should be inclined to attribute them to the last, in consequence of similar attacks both in infants and adults, at that period being somewhat epidemic.

I shall take the liberty of troubling you with another case of the Cow-pox, from the circumstance of its being particularly striking in resisting the contagion of Small-pox.

I was desired to attend the son of Mr. Prosser, of Holborn, who on the following day had an eruption, which proved to be the *small-pox*, and that of the confluent kind, attended in the secondary fever with delirium and very slight hopes of recovery. This child was reported to have had the small-pox three years before, when on a visit in the country. Mr. P. had an apprentice, whom he and myself endeavoured to persuade to receive the Vaccine protection by inoculation, but without effect, till the eruption of small-pox had made its appearance on the son nine days, at which it was at its crisis; he then consenting, I inserted fresh Vaccine fluid into the arm of the apprentice, *(who till this period had always been in the same room with the son, and* SLEPT *with him)* without hope of its having the desired effect; the arm shewed marks of inflammation on the third day, and was very evident on the fourth, continuing regular in its progress, producing the characteristic areola as well as soreness in the axilla. I observed on the eighth day the pustule did not look favorable, and I suspected pected it had been rubbed; but was informed the boy had seen his mother the day before, who having a strong aversion to the Cow-pox, had prevailed on her son to endeavour to eradicate it, which the boy was seen by his mistress attempting to do soon after he went to bed, by picking open the pustule with a pin. The two lads continued living in the same room together till the present time, a period of more than three months; the son recovered, and the apprentice was not affected by actual contact with the small-pox patient. I am, &c.

*Tooley Street, April* 20, 1803. I. F. WAGSTAFFE.

